# 4-Chloro-*N*-[(*E*)-2,4-dichloro­benzyl­idene]aniline

**DOI:** 10.1107/S1600536810035774

**Published:** 2010-09-11

**Authors:** Umar Hayat, Waseeq Ahmad Siddiqui, M. Nawaz Tahir, Ghulam Hussain

**Affiliations:** a Department of Chemistry, University of Sargodha, Sargodha, Pakistan; b Department of Physics, University of Sargodha, Sargodha, Pakistan

## Abstract

In the mol­ecule of the title compound, C_13_H_8_Cl_3_N, the 4-chloro­aniline and 2,4-dichloro­benzaldehyde moieties are planar with r.m.s. deviation of 0.0115 and 0.0116 Å, respectively, and are oriented at a dihedral angle of 13.94 (8)°.

## Related literature

For related structures, see: Bernstein (1972 ▶), Yin *et al.* (2007 ▶). For graph-set notation, see: Bernstein *et al.* (1995 ▶).
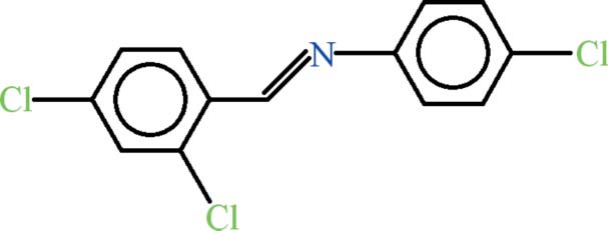


## Experimental

### 

#### Crystal data

C_13_H_8_Cl_3_N
*M* *_r_* = 284.55
Monoclinic, 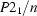

*a* = 3.9665 (3) Å
*b* = 27.639 (2) Å
*c* = 11.4287 (9) Å
β = 99.165 (3)°
*V* = 1236.93 (16) Å^3^
*Z* = 4
Mo *K*α radiation
μ = 0.71 mm^−1^
*T* = 296 K
0.32 × 0.12 × 0.08 mm

#### Data collection

Bruker Kappa APEXII CCD diffractometer
Absorption correction: multi-scan (*SADABS*; Bruker, 2005 ▶) *T*_min_ = 0.903, *T*_max_ = 0.946
9236 measured reflections
2239 independent reflections
1372 reflections with *I* > 2σ(*I*)
*R*_int_ = 0.047

#### Refinement

*R*[*F* ^2^ > 2σ(*F* ^2^)] = 0.047
*wR*(*F* ^2^) = 0.107
*S* = 1.02
2239 reflections
154 parameters
H-atom parameters constrained
Δρ_max_ = 0.20 e Å^−3^
Δρ_min_ = −0.21 e Å^−3^

### 

Data collection: *APEX2* (Bruker, 2009 ▶); cell refinement: *SAINT* (Bruker, 2009 ▶); data reduction: *SAINT*; program(s) used to solve structure: *SHELXS97* (Sheldrick, 2008 ▶); program(s) used to refine structure: *SHELXL97* (Sheldrick, 2008 ▶); molecular graphics: *ORTEP-3 for Windows* (Farrugia, 1997 ▶) and *PLATON* (Spek, 2009 ▶); software used to prepare material for publication: *WinGX* (Farrugia, 1999 ▶) and *PLATON*.

## Supplementary Material

Crystal structure: contains datablocks global, I. DOI: 10.1107/S1600536810035774/rz2483sup1.cif

Structure factors: contains datablocks I. DOI: 10.1107/S1600536810035774/rz2483Isup2.hkl

Additional supplementary materials:  crystallographic information; 3D view; checkCIF report

## Figures and Tables

**Fig. 1.**
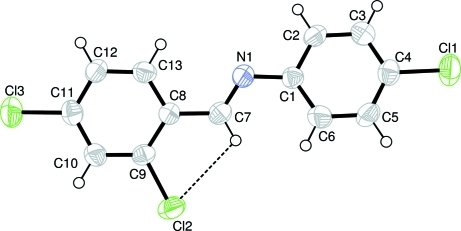
View of the title compound with the atom numbering scheme. The thermal ellipsoids are drawn at the 50% probability level. H-atoms are shown as small spheres of arbitrary radii. The dotted line represent the intramolecular H-bonds.

## supplementary crystallographic information

### Comment

The synthesis and crystal structure of the title compound is herein reported as a part of our new project aimed to the study of new Schiff bases of 2,4-dichlorobenzaldehyde and their metal complexation abilities. The crystal structures of the related compounds *N*-(2,4-dichlorobenzylidene)aniline (Bernstein, 1972) and *N*-(2,4-dichlorobenzylidene)-*N'*-phenylhydrazine (Yin *et al.*, 2007) have been already published.

In the title compound (Fig. 1), the 4-chloroaniline (C1—C6/N1/Cl1) and 2,4-dichlorobenzaldehyde (C7—C13/Cl2/Cl3) moieties are planar with r. m. s. deviation of 0.0115 and 0.0116 Å, respectively. The dihedral angle between the two moieties is 13.94 (8)°. There exist a weak intramolecular C—H···Cl hydrogen bond (Table 1, Fig. 1) forming an *S*(5) ring motif (Bernstein *et al.*, 1995). The crystal structure is stabilized only by van der Waals interactions.

### Experimental

An equimolar ratio of 2,4-dichlorobanzaldehyde and 4-chloroaniline were refluxed in xylene (20 ml) with acetic acid (2 ml) as a catalyst for an hour. The completion of the reaction was monitored through TLC. After completion of the reaction, the xylene was distilled out and the solid product obtained was dried. The dried crude material obtained was recrystallized in ethyl acetate and methanol (1:1 *v*/*v*) to affoard light pink needles of the title compound in 24 h.

### Refinement

The H-atoms were positioned geometrically (C–H = 0.93 Å) and were included in the refinement in the riding model approximation, with with *U*_iso_(H) = 1.2*U*_eq_(C).

### Crystal data

**Table:**

C_13_H_8_Cl_3_N	*F*(000) = 576
*M**_r_* = 284.55	*D*_x_ = 1.528 Mg m^−^^3^
Monoclinic, *P*2_1_/*n*	Mo *K*α radiation, λ = 0.71073 Å
Hall symbol: -P 2yn	Cell parameters from 1372 reflections
*a* = 3.9665 (3) Å	θ = 2.0–25.2°
*b* = 27.639 (2) Å	µ = 0.71 mm^−^^1^
*c* = 11.4287 (9) Å	*T* = 296 K
β = 99.165 (3)°	Needles, light pink
*V* = 1236.93 (16) Å^3^	0.32 × 0.12 × 0.08 mm
*Z* = 4	

### Data collection

**Table:**

Bruker Kappa APEXII CCD diffractometer	2239 independent reflections
Radiation source: fine-focus sealed tube	1372 reflections with *I* > 2σ(*I*)
graphite	*R*_int_ = 0.047
Detector resolution: 8.10 pixels mm^-1^	θ_max_ = 25.2°, θ_min_ = 2.0°
ω scans	*h* = −4→4
Absorption correction: multi-scan (*SADABS*; Bruker, 2005)	*k* = −32→33
*T*_min_ = 0.903, *T*_max_ = 0.946	*l* = −13→13
9236 measured reflections	

### Refinement

**Table:**

Refinement on *F*^2^	Primary atom site location: structure-invariant direct methods
Least-squares matrix: full	Secondary atom site location: difference Fourier map
*R*[*F*^2^ > 2σ(*F*^2^)] = 0.047	Hydrogen site location: inferred from neighbouring sites
*wR*(*F*^2^) = 0.107	H-atom parameters constrained
*S* = 1.02	*w* = 1/[σ^2^(*F*_o_^2^) + (0.032*P*)^2^ + 0.7441*P*] where *P* = (*F*_o_^2^ + 2*F*_c_^2^)/3
2239 reflections	(Δ/σ)_max_ < 0.001
154 parameters	Δρ_max_ = 0.20 e Å^−^^3^
0 restraints	Δρ_min_ = −0.21 e Å^−^^3^

### Special details

**Table:**

Geometry. Bond distances, angles *etc*. have been calculated using the rounded fractional coordinates. All su's are estimated from the variances of the (full) variance-covariance matrix. The cell e.s.d.'s are taken into account in the estimation of distances, angles and torsion angles
Refinement. Refinement of *F*^2^ against ALL reflections. The weighted *R*-factor *wR* and goodness of fit *S* are based on *F*^2^, conventional *R*-factors *R* are based on *F*, with *F* set to zero for negative *F*^2^. The threshold expression of *F*^2^ > σ(*F*^2^) is used only for calculating *R*-factors(gt) *etc*. and is not relevant to the choice of reflections for refinement. *R*-factors based on *F*^2^ are statistically about twice as large as those based on *F*, and *R*- factors based on ALL data will be even larger.

### Fractional atomic coordinates and isotropic or equivalent isotropic displacement parameters (Å^2^)

**Table:**

	*x*	*y*	*z*	*U*_iso_*/*U*_eq_	
Cl1	−0.1794 (3)	0.20049 (4)	−0.09024 (9)	0.0739 (4)	
Cl2	1.1913 (3)	0.18426 (3)	0.60890 (9)	0.0635 (4)	
Cl3	1.3135 (3)	0.02321 (4)	0.87154 (8)	0.0702 (4)	
N1	0.4889 (7)	0.10444 (10)	0.3494 (2)	0.0458 (10)	
C1	0.3428 (8)	0.12998 (12)	0.2462 (3)	0.0409 (11)	
C2	0.1799 (9)	0.10234 (13)	0.1521 (3)	0.0521 (14)	
C3	0.0224 (9)	0.12381 (14)	0.0490 (3)	0.0567 (16)	
C4	0.0238 (8)	0.17295 (14)	0.0389 (3)	0.0489 (14)	
C5	0.1799 (9)	0.20122 (14)	0.1310 (3)	0.0576 (14)	
C6	0.3342 (9)	0.17971 (14)	0.2341 (3)	0.0573 (12)	
C7	0.7186 (8)	0.12440 (13)	0.4242 (3)	0.0440 (12)	
C8	0.8657 (7)	0.10042 (12)	0.5344 (3)	0.0383 (11)	
C9	1.0827 (8)	0.12398 (12)	0.6247 (3)	0.0409 (11)	
C10	1.2154 (8)	0.10111 (13)	0.7292 (3)	0.0469 (12)	
C11	1.1380 (8)	0.05286 (13)	0.7431 (3)	0.0469 (14)	
C12	0.9276 (9)	0.02799 (13)	0.6552 (3)	0.0494 (12)	
C13	0.7960 (8)	0.05201 (13)	0.5530 (3)	0.0467 (12)	
H2	0.17733	0.06882	0.15891	0.0624*	
H3	−0.08427	0.10490	−0.01344	0.0681*	
H5	0.18079	0.23473	0.12349	0.0691*	
H6	0.43465	0.19895	0.29688	0.0683*	
H7	0.79497	0.15515	0.40831	0.0528*	
H10	1.35406	0.11780	0.78919	0.0562*	
H12	0.87615	−0.00443	0.66514	0.0591*	
H13	0.65459	0.03524	0.49395	0.0560*	

### Atomic displacement parameters (Å^2^)

**Table:**

	*U*^11^	*U*^22^	*U*^33^	*U*^12^	*U*^13^	*U*^23^
Cl1	0.0774 (7)	0.0752 (8)	0.0638 (7)	0.0095 (6)	−0.0051 (5)	0.0237 (6)
Cl2	0.0658 (6)	0.0420 (6)	0.0792 (7)	−0.0104 (5)	0.0009 (5)	0.0039 (5)
Cl3	0.0822 (7)	0.0674 (8)	0.0552 (6)	0.0117 (6)	−0.0065 (5)	0.0141 (5)
N1	0.0531 (18)	0.0406 (18)	0.0431 (16)	0.0013 (14)	0.0058 (13)	0.0019 (14)
C1	0.0409 (19)	0.041 (2)	0.0413 (19)	−0.0013 (16)	0.0077 (15)	0.0013 (16)
C2	0.070 (3)	0.035 (2)	0.050 (2)	−0.0030 (18)	0.0056 (18)	0.0025 (17)
C3	0.066 (3)	0.052 (3)	0.049 (2)	−0.004 (2)	0.0000 (18)	−0.0037 (19)
C4	0.043 (2)	0.053 (3)	0.051 (2)	0.0061 (18)	0.0088 (16)	0.0085 (19)
C5	0.058 (2)	0.037 (2)	0.074 (3)	0.0060 (19)	−0.001 (2)	0.007 (2)
C6	0.060 (2)	0.046 (2)	0.061 (2)	0.0027 (19)	−0.0057 (19)	−0.007 (2)
C7	0.043 (2)	0.043 (2)	0.048 (2)	−0.0034 (16)	0.0137 (16)	0.0043 (17)
C8	0.0338 (18)	0.040 (2)	0.0417 (19)	0.0008 (15)	0.0078 (14)	0.0022 (16)
C9	0.0379 (19)	0.037 (2)	0.049 (2)	−0.0018 (15)	0.0105 (15)	−0.0006 (16)
C10	0.043 (2)	0.048 (2)	0.047 (2)	0.0007 (17)	−0.0011 (15)	−0.0010 (18)
C11	0.045 (2)	0.053 (3)	0.042 (2)	0.0076 (18)	0.0049 (16)	0.0015 (18)
C12	0.061 (2)	0.040 (2)	0.047 (2)	0.0003 (18)	0.0084 (17)	0.0054 (17)
C13	0.051 (2)	0.044 (2)	0.045 (2)	−0.0051 (17)	0.0074 (16)	−0.0060 (17)

### Geometric parameters (Å, °)

**Table:**

Cl1—C4	1.740 (4)	C8—C13	1.390 (5)
Cl2—C9	1.738 (3)	C9—C10	1.379 (5)
Cl3—C11	1.728 (4)	C10—C11	1.383 (5)
N1—C1	1.417 (4)	C11—C12	1.382 (5)
N1—C7	1.272 (4)	C12—C13	1.372 (5)
C1—C2	1.392 (5)	C2—H2	0.9300
C1—C6	1.381 (5)	C3—H3	0.9300
C2—C3	1.377 (5)	C5—H5	0.9300
C3—C4	1.363 (5)	C6—H6	0.9300
C4—C5	1.376 (5)	C7—H7	0.9300
C5—C6	1.374 (5)	C10—H10	0.9300
C7—C8	1.460 (5)	C12—H12	0.9300
C8—C9	1.395 (5)	C13—H13	0.9300
			
Cl2···C7^i^	3.602 (4)	C13···C12^viii^	3.547 (5)
Cl3···Cl3^ii^	3.3276 (14)	C13···C10^vii^	3.561 (5)
Cl1···H6^iii^	3.1300	C6···H7	2.5700
Cl1···H10^iv^	3.1200	C7···H6	2.6700
Cl2···H5^v^	3.0400	C12···H13^viii^	3.1000
Cl2···H5^vi^	2.9500	C13···H13^ix^	3.0000
Cl2···H7	2.6900	H5···Cl2^x^	2.9500
N1···C7^vii^	3.347 (4)	H5···Cl2^iii^	3.0400
N1···H13	2.5400	H6···C7	2.6700
C1···C7^vii^	3.449 (5)	H6···H7	2.1300
C7···C1^i^	3.449 (5)	H6···Cl1^v^	3.1300
C7···Cl2^vii^	3.602 (4)	H7···Cl2	2.6900
C7···N1^i^	3.347 (4)	H7···C6	2.5700
C8···C9^vii^	3.487 (4)	H7···H6	2.1300
C9···C8^i^	3.487 (4)	H10···Cl1^xi^	3.1200
C10···C13^i^	3.561 (5)	H13···N1	2.5400
C11···C12^i^	3.506 (5)	H13···C12^viii^	3.1000
C12···C13^viii^	3.547 (5)	H13···C13^ix^	3.0000
C12···C11^vii^	3.506 (5)	H13···H13^ix^	2.3200
			
C1—N1—C7	119.9 (3)	Cl3—C11—C12	119.8 (3)
N1—C1—C2	116.6 (3)	C10—C11—C12	121.0 (3)
N1—C1—C6	125.4 (3)	C11—C12—C13	118.8 (3)
C2—C1—C6	117.9 (3)	C8—C13—C12	122.6 (3)
C1—C2—C3	121.1 (3)	C1—C2—H2	119.00
C2—C3—C4	119.6 (3)	C3—C2—H2	119.00
Cl1—C4—C3	120.0 (3)	C2—C3—H3	120.00
Cl1—C4—C5	119.4 (3)	C4—C3—H3	120.00
C3—C4—C5	120.6 (3)	C4—C5—H5	120.00
C4—C5—C6	119.7 (4)	C6—C5—H5	120.00
C1—C6—C5	121.1 (3)	C1—C6—H6	119.00
N1—C7—C8	121.9 (3)	C5—C6—H6	119.00
C7—C8—C9	122.7 (3)	N1—C7—H7	119.00
C7—C8—C13	120.5 (3)	C8—C7—H7	119.00
C9—C8—C13	116.8 (3)	C9—C10—H10	121.00
Cl2—C9—C8	120.3 (3)	C11—C10—H10	121.00
Cl2—C9—C10	117.6 (3)	C11—C12—H12	121.00
C8—C9—C10	122.1 (3)	C13—C12—H12	121.00
C9—C10—C11	118.7 (3)	C8—C13—H13	119.00
Cl3—C11—C10	119.2 (3)	C12—C13—H13	119.00
			
C7—N1—C1—C2	160.2 (3)	N1—C7—C8—C13	9.7 (5)
C7—N1—C1—C6	−23.3 (5)	C7—C8—C9—Cl2	−0.4 (4)
C1—N1—C7—C8	177.2 (3)	C7—C8—C9—C10	178.9 (3)
N1—C1—C2—C3	178.4 (3)	C13—C8—C9—Cl2	178.9 (2)
C6—C1—C2—C3	1.6 (5)	C13—C8—C9—C10	−1.8 (5)
N1—C1—C6—C5	−178.6 (3)	C7—C8—C13—C12	−179.8 (3)
C2—C1—C6—C5	−2.2 (5)	C9—C8—C13—C12	0.9 (5)
C1—C2—C3—C4	−0.4 (5)	Cl2—C9—C10—C11	−178.8 (3)
C2—C3—C4—Cl1	−179.1 (3)	C8—C9—C10—C11	1.9 (5)
C2—C3—C4—C5	−0.3 (5)	C9—C10—C11—Cl3	177.9 (3)
Cl1—C4—C5—C6	178.6 (3)	C9—C10—C11—C12	−1.0 (5)
C3—C4—C5—C6	−0.3 (5)	Cl3—C11—C12—C13	−178.8 (3)
C4—C5—C6—C1	1.6 (5)	C10—C11—C12—C13	0.1 (5)
N1—C7—C8—C9	−171.0 (3)	C11—C12—C13—C8	−0.1 (5)

Symmetry codes: (i) *x*+1, *y*, *z*; (ii) −*x*+3, −*y*, −*z*+2; (iii) *x*−1/2, −*y*+1/2, *z*−1/2; (iv) *x*−2, *y*, *z*−1; (v) *x*+1/2, −*y*+1/2, *z*+1/2; (vi) *x*+3/2, −*y*+1/2, *z*+1/2; (vii) *x*−1, *y*, *z*; (viii) −*x*+2, −*y*, −*z*+1; (ix) −*x*+1, −*y*, −*z*+1; (x) *x*−3/2, −*y*+1/2, *z*−1/2; (xi) *x*+2, *y*, *z*+1.

### Hydrogen-bond geometry (Å, °)

**Table:**

*D*—H···*A*	*D*—H	H···*A*	*D*···*A*	*D*—H···*A*
C7—H7···Cl2	0.93	2.69	3.074 (4)	106
